# Lagging and Flagging: Air Pollution, Shale Gas Exploration and the Interaction of Policy, Science, Ethics and Environmental Justice in England

**DOI:** 10.3390/ijerph17124320

**Published:** 2020-06-17

**Authors:** Andrew Watterson, William Dinan

**Affiliations:** 1 Occupational and Environmental Health Research Group, Faculty of Health Sciences, University of Stirling, Stirling FK9 4LA, Scotland, UK; 2 Communications, Media & Culture, Faculty of Arts & Humanities, University of Stirling, Stirling FK9 4LA, Scotland, UK; william.dinan1@stir.ac.uk

**Keywords:** shale exploration, air pollution, ethics, environmental justice

## Abstract

The science on the effects of global climate change and air pollution on morbidity and mortality is clear and debate now centres around the scale and precise contributions of particular pollutants. Sufficient data existed in recent decades to support the adoption of precautionary public health policies relating to fossil fuels including shale exploration. Yet air quality and related public health impacts linked to ethical and environmental justice elements are often marginalized or missing in planning and associated decision making. Industry and government policies and practices, laws and planning regulations lagged well behind the science in the United Kingdom. This paper explores the reasons for this and what shaped some of those policies. Why did shale gas policies in England fail to fully address public health priorities and neglect ethical and environmental justice concerns. To answer this question, an interdisciplinary analysis is needed informed by a theoretical framework of how air pollution and climate change are largely discounted in the complex realpolitik of policy and regulation for shale gas development in England. Sources, including official government, regulatory and planning documents, as well as industry and scientific publications are examined and benchmarked against the science and ethical and environmental justice criteria. Further, our typology illustrates how the process works drawing on an analysis of official policy documents and statements on planning and regulatory oversight of shale exploration in England, and material from industry and their consultants relating to proposed shale oil and gas development. Currently the oil, gas and chemical industries in England continue to dominate and influence energy and feedstock-related policy making to the detriment of ethical and environmental justice decision making with significant consequences for public health.

## 1. Introduction

The WHO Europe in 2013 used an expert scientific group to review European policies on air pollution from a health perspective [1]. The review concluded that a considerable amount of new scientific information on the adverse effects on health of particulate matter, ozone and nitrogen dioxide, observed at levels commonly present in Europe, reinforced the scientific conclusions of the WHO air quality guidelines, updated in 2005, indicating that adverse effects could occur from air pollution concentrations lower than those in guidelines published at the time. It also provided scientific arguments for taking decisive actions to improve air quality and reduce the burden of disease associated with air pollution in Europe. The findings were not taken up across the region at that stage. 

In the USA, oil and chemical companies’ efforts to influence policy makers and cast doubt on research findings on the cause and effects of air pollution have been detailed over many years [2,3]. The industry has used doubt as a strategy to delay actions on hazardous substances linked to air pollution and on specific industries, particularly the oil and gas industries [3]. Toxic crimes due to chemical exposures have often been ignored [4,5]. Yet detailed reports of the health effects of air pollution have been available since at least the late 1990s and early 2000s, and often long before [6,7]. Table 1 highlights some of the dates of knowledge on air pollution and ill-health effects and the toll this takes on public health. It serves as an illustration of how slow policy and regulations are in addressing the public health problems produced by air pollution.

In 2008, the WHO issued guidelines on air quality, with evidence from a range of sources. These are recognized as the international benchmark for setting air quality standards of 10 μg/m^3^ for PM2.5 [1]. The UK’s Department for Environment, Food and Rural Affairs (DEFRA) air quality documents acknowledge that “It is possible that very sensitive individuals may experience health effects even on low air pollution days” [8,9]. The guidance further states: “It is known that, when levels of air pollutants rise, adults suffering from heart conditions, and adults and children with lung conditions, are at increased risk of becoming ill and needing treatment” [8].

Despite recognition in official documents and in guidelines published by the WHO, connecting science, policy and practice raises questions about whether the science (and gaps in the science) along with environmental justice and ethics are being ignored in practice [10,11,12,13,14]. Our paper explores the tension between science and politics in the context of contested development—in this case, shale exploration in the UK, with a focus on England. We highlight the contradictions between efforts by government in the UK to secure the acceptance of shale development by relying on claims that policy is led by evidence, while in practice ignoring important new scientific findings and a burgeoning evidence base that challenged some of the key assumptions of preferred government policy. While the idea that policy making should be led, or at least informed, by evidence is contested by some, and considered a chimera by others, we do hold to the principle that science can positively contribute to policy deliberation, knowledge and the wider public good. We will return to this argument in the discussion below as we synthesize the evidence from our typology, our analytic framework, and the existing relevant scientific evidence base in relation to shale oil and gas development and public health. This would now include questions about assessments of both past and proposed shale exploration and their environmental health impacts [15]. Tighter limits on air pollutants in the UK were called for in 2019 to reduce dangers of heart disease, cancers and poor brain development in children. There were calls for Europe (including the UK) to introduce tougher standards than the existing ultrafine particle standard of 25 µg per cubic metre. “Particles are a major and invisible danger to our health, especially in London and our big cities. The US has a standard of 12 micrograms of ultra-fine particles per cubic metre, while the WHO standard is 10 micrograms” [16]. 

**Table 1:** Chronology on recognition of air pollution and its effects—UK, unless otherwise stated.

Date	Effects	Source
400 BC	Hippocrates associated the city with air pollution in Greece	Heidorn 1978 [7]
1	Industrial air pollution recorded in Tyre, Lebanon	Heidorn 1978
852	London noted its foul air	Heidorn 1978
1257	Nottingham found too smoky and therefore uninhabitable	Heidorn 1978
1273	1st smoke abatement law enacted in London prohibiting use of coal as “prejudicial to health”	Heidorn 1978
1307	Edward I prohibits coal use because of pollution that could not be ameliorated	Heidorn 1978
1661	John Evelyn published the first significant study on air pollution in London	Heidorn 1978
1662	John Graunt analysed Bills of Mortality, using health statistics to speculate that much of London’s public health problem was due to air pollution	Heidorn 1978
1875	Public Health Act included the need for smoke abatement	Heidorn 1978
1933	Ashworth published a study of pollution in Manchester	Heidorn 1978
1937–1939	Major survey of atmospheric pollution in Leicester	Heidorn 1978
1952	London smog estimated in 1970s to have killed 4000 people. Suspended particulates of 400–4500 µg/m^3^ are reported	Heidorn 1978
1975	European Community (EC) Directive 75/716/EEC: on sulphur content of two types of gas oil (diesel and heating oil)	Enviropedia nd [6]
1980	EC Directive 80/779/EEC: air quality limit values and guide values for sulphur dioxide and suspended particles	Enviropedia nd
1988	1988—EC Directive 88/609/EEC: limited emissions of SO2 and NOx and particulates from power stations and other large combustion plants	Enviropedia nd
2008	UK air pollution mortality estimated at 29,000 deaths, equivalent to associated loss of 340,000 life years	COMEAP 2010 [10]
2016	Annual mortality burden in the UK from exposure to outdoor air pollution is equivalent to approximately 40,000 deaths	RCP 2016 [11]
2018	Long-term exposure to human-made air pollution in the UK estimated to have an annual impact on shortening lifespans, equivalent to 28,000 to 36,000 deaths	COMEAP 2018 [12]

Against this background, studies modelling air pollution did find that UK mortality declined between 1970 and 2010 and UK-attributable mortality due to exposure to PM2.5 and NO_2_ was estimated to have declined by 56% and 44% but ozone-attributable respiratory mortality increased by 17% [17]. The authors noted that air pollution still posed significant risks to UK public health. The Royal College of Physicians (RCP) and the UK government continue to voice concerns about the current estimates of mortality from poor air quality. More recent research indicates the air pollution risks from PM2.5 both in the short term and long term may be wider than hitherto known. For example, findings from a 2019 study support the hypothesis of a link between long-term PM2.5 exposure and depression, as well as suggesting possible links with anxiety and between short-term PM10 exposure and suicide [18]. Major concerns about current and future NOx levels in the UK are also part of this air pollution issue culture [19]. Moreover, most studies usually do not consider climate change impacts of air pollution or any ethical and environmental justice issues raised by the statistics. Such questions, if they are pursued, can be found in political and social science literature. The inability or unwillingness to meaningfully factor such concerns, and their evidentiary underpinning, into planning, impact assessments and regulatory decision making remains a particular blindspot in the UK’s governance of environment and public health.

Air pollution has many causes and fossil fuels are one important factor among several but add to the load in terms of their product and its extraction [20]. The UN also had significant reservations about the safety of shale exploration [21]. The major air pollution hazards of shale oil and gas have been documented for several years as Table 2 shows.

As early as 2012, a US study examined in some depth issues around shale gas emissions and possible human risks [23]. In 2018, UK researchers’ models suggest that the potential impacts of shale gas air emissions, through volatile organic compounds and oxides of nitrogen, on UK air quality and human health should be highly controlled to prevent adverse health impacts [24]. There may be uncertainty and disagreement about some shale exploration environmental impacts that can undermine trust in scientists and government officials [25]. However, in terms of impacts on climate change and how both the construction and operation of shale oil and gas sites will add to air pollution, there is no dispute now but only debate on the level of impact. While there is some uncertainty about the magnitude of these effects in both the short and medium term, the point to note here is that this uncertainty is politically exploitable in principle by those with interests who might wish to argue for no policy or regulatory response to the latest research findings and data models. Air pollution researchers have observed that the harder they look, the more they find about the adverse health effects of fine particulate air pollution. They warn ‘we should not mistake knowledge gaps for paucity of evidence’ ([26], p. 2). One of the threats to public health has been the application of a ‘don’t look, don’t find, no problem’ style of policy and regulation. In England, this now means there is an increasing divide between planning law, government policy and what is needed to protect public health from air pollution based on the science [27,28]. 

A small number of researchers have specifically looked at fracking in terms of environmental justice and produced a range of analyses [29]. Few engineers have ventured into the field [30]. The ethics of environmental decision making, which is relevant to shale exploration, has been almost entirely neglected in UK government policy documents. Only a handful of publications about shale oil and gas and ethics have emanated from the UK and a similar number in the US [31,32,33,34]. These rarely or only briefly address air pollution issues, although several do focus on water pollution and fracking from an ethical standpoint. Cotton observed that in the UK “only by ‘re-localising’ the scale of fracking governance can political equality be ensured and the distributive and procedural environmental injustices be ameliorated” [35]. Researchers have addressed issues of climate and intergenerational justice, topics entirely missing from several key UK government and official fracking reports [36,37,38,39].

## 2. Materials and Methods 

This paper uses empirical and theoretical approaches to environmental justice and ethics adopted or advocated by researchers and professional bodies relevant to shale exploration air pollution issues [2,3,40,41,42,43,44]. It deliberately does not provide a literature review of shale oil and gas air pollution studies but rather draws on air pollution studies that help to inform an understanding of how environmental justice and ethical issues have been addressed by government, industry and professional bodies. A Gramscian framing is used [45,46] which foregrounds the political economy of shale exploration. Our analysis, drawing on Gramscian concepts of hegemony and ‘war of position’ seeks to unpack how a particular issue—in this case, fracking—comes to be defined over time and how that definitional work occurs in a variety of fora, including policy, regulatory, civil society and media settings and spanning expert and public discourses [47]. Our analytic approach offers a reading of official documents and policy papers that is alive to how the wider politics of shale exploration may be reflected in official communications about this subject. It is sensitive to how scientific information and data may be systematically organised in and out of official documents. Official documents are not simply neutral documents, they are produced for a purpose. Data do not simply speak for themselves in policy networks and deliberation. How scientific evidence and data are contextualised and framed in policy documents is therefore both a topic and a resource for this study. We have focused on the particular issues of the risks of air pollution across the fracking lifecycle. We examine the key English and Scottish official documents and industry reports that shaped UK shale exploration public health policy. The official papers and reports we review can be considered the obligatory reference points for UK policy makers in this field, evidenced by their repeated and consistent citation in government, industry, legal and consultancy papers on shale exploration. The majority of these reports and papers also cite and cross-reference each other in a policy loop and frequently exclude other extant but critical papers on the effects of shale oil and gas development. The evidence base for critiquing these papers has been included in our previous reports and peer reviewed papers cited in the discussion below. One major literature review paper on health was also included for comparison with these key papers and reports. This allowed us to explore how public health issues are framed and positioned in policy discourse and practice.

The typology we have developed focuses on how public health issues and inequalities, environmental justice and ethical concerns have been featured in planning, regulatory and industry analyses of shale development and why. It suggests that rather than policy and regulation being ‘led by the science’, there appears to be a determined effort to ignore some of the emerging scientific evidence on the risks and harms of air pollution linked to shale development. In our usage, ‘war of position’ refers to the building of alliances and coalitions of different interests and identities to secure political compliance and practical dominance identified in our diagram. It can be thought of as the long-term cultural struggle to secure ideological supremacy, or hegemony. This approach is sensitive to the different interests, coalitions and strategic alliances that politically contest an issue and discursively shape an issue culture. For those issues that rely on scientific expertise, the role of experts and regulators becomes politically and decisionally salient. 

Our focus in this paper is therefore on industry, regulators, consultants and planners and seeks to examine how these actors choose and use scientific evidence to warrant their preferred solutions and judgements about the likely impacts of shale exploration. Reviewing how shale development has been promoted, deliberated and regulated in the UK suggests that industry, their consultants, and key departments of the UK government share a similar understanding of shale development, which emphasises the economic benefits of exploration. In Scotland, the political dynamics are quite different: industry does not enjoy the active support of key ministries and the Scottish government has adopted a more precautionary approach that appears to weigh environmental and public health issues more seriously than the UK government as we evidence later. A core question raised in our analysis is how the concerns of industry appear to influence the UK government superstructure, local authorities and planners so effectively [37]. The analysis therefore draws on a range of key government, industry and professional reports that have investigated shale exploration.

The framing of the analysis is described in Diagram 1. We therefore explore how the public health and related environmental justice and ethical dimensions were either marginalised or ignored in both national English shale oil and gas policy making and particular project developments.
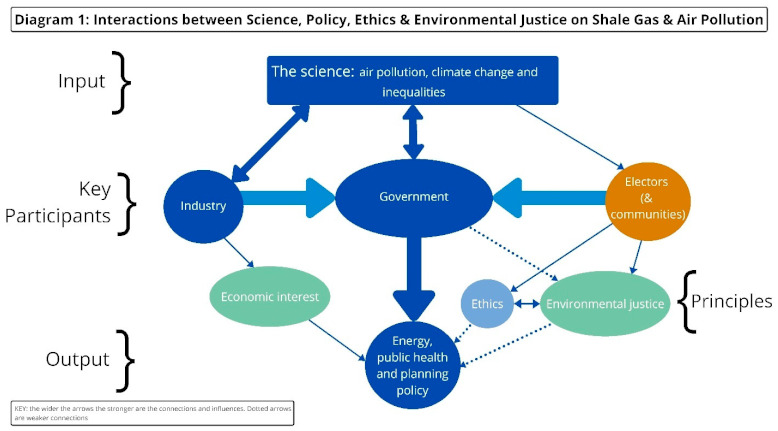


This is carried out firstly through a section exploring the science on air pollution and shale exploration. The papers and reports cited in this section are usually neither adequately addressed nor debated in the context of environmental justice or ethics by industry or English policy makers and planners. It is not assumed that they cannot be contested but in the context in which our analysis is applied, they remain absent from much of the policy and practice domain. Secondly, we have examined the role of health impact assessments and their ethical underpinning or absence when applied to planning, policy and environmental statement documentation. Within this field, it is necessary to identify and discuss specific strands relating to inequalities and environmental justice in shale oil and gas developments. Thirdly, an analysis was carried out of key government and industry-funded shale reports to test to what extent they too addressed environmental justice and ethical questions relating to how shale exploration would or could affect air pollution and impact on health inequalities. Fourthly, a typology is presented which offers a concise summary of the major arguments presented by industry and planners. Those arguments are reflected in government policies and regulations, in English enquiries as to why the impacts of air pollution on environmental justice and hence health inequalities should be ignored. We draw out some of the key assumptions and preferences that underpin this policy framework and identify how these articulate with the latest scientific evidence, models and expert consensus. This typology highlights the ethical implications for public health policy which are sketched in the final discussion section and conclusions.

## 3. Results

### 3.1. Situating the Science on Air Pollution and Shale Exploration

There is frequently a major mismatch between what scientific research flags up and how quickly regulators, industry and their consultants catch up with, cite and use that science. At what point (if at all) do governments factor new research into legislation and policies, and to what extent do planners and regulators take the latest research-based evidence into account? 

The skewing of research and the manipulation of scientific findings both generally and within the public health fields by industry have been well documented over many decades [3,48,49]. Some of the efforts to distort how scientific evidence is weighed on issues where corporate interests and the wider public interest collide are conducted under the banner of ‘sound science’ [50]. Another dimension to the disputation of scientific evidence has been the corporate assault on the precautionary principle, and in the European context, recent efforts to promote the innovation principle as core to policy and regulatory decision making. The analysis of the emergence and strategies of the climate denial movement point to the contestation of science as a key aspect of their political influence [47,51,52,53,54]. Attacks on individual scientists also appear to be orchestrated. This fits into a wider pattern of organised strategic communication, where corporations are active in projecting and protecting their interests in policy circles, expert networks, public fora and in judicial setting like public hearings and the courts [54,55]. Our Gramscian approach is sensitive to how ‘common sense’ about issues of science and public health are forged over time and across a variety of settings and arenas. The evidence also suggests that corporations enjoy a structural advantage in having the resources to invest and engage in a definitional war of position to secure its interests. In relation to shale exploration, these resources include lobbying and public affairs expertise, both in-house and hired consultants. It also includes those who work on behalf of corporations in regulatory affairs and in managing planning applications, spanning the environmental, health and safety, as well as managing local community relations through public relations activities—all of which is geared towards securing consent for shale developments. 

These studies are equally relevant to the shale oil and gas industry and sometimes relate to companies that operate or invest in shale exploration [56,57,58]. In recent years, there has been growing concern about how the fossil fuel industry can influence the framing and processing of energy development at a range of levels: central and local government and including legal and planning mechanisms. Our focus here is not on the spread of misinformation or disinformation on scientific issues. The concern is to explore how presumably scientifically literate and informed experts and regulators are party to regulatory and policy outcomes that apparently contradict or ignore scientific consensus. There is some research which points to the role of governments (in Australia and Canada) in denying science, especially in relation to climate issues, referred to as ‘ignorance building’ [59]. Other research considers ‘ignorance as a necessary social achievement rather than as a simple background failure to acquire, store and retrieve knowledge’ ([60], p. 107), suggesting that ignorance may be necessary for organisations, especially those dealing with ‘wicked problems’ associated with environment and public health. Industry reappears in much of the literature on contesting science [61]. 

“Protecting the public’s health, preventing disease, and promoting well-being must be the unambiguous goals of research in occupational and environmental health” ([62], p. 1). The hazards of air pollution linked to unconventional oil and gas development have been well documented, sometimes along with likely risks to public health of very low-level exposures [63,64,65,66,67]. Potential risks from shale in Europe have been identified [68]. Air concentrations of volatile compounds near oil and gas production facilities have been flagged specifically in a community context [69]. Human exposure to unconventional natural gas development has been noted with periodic high exposure to chemical mixtures in ambient air [70]. Other studies have looked at particular air pollutants from shale oil and gas facilities and winter haze around shale sites [71,72]. One study using geographic information assessments of maternal ambient health hazards and adverse birth outcomes in Canada included oil and gas well pads. [73].

US and UK reviews on public health in several publications beyond central government agencies in Europe and US have also looked at air pollution and shale developments [64,74,75,76]. Leading environmental and medical experts have flagged shale exploration as a major threat to public health including air pollution linked to climate change [77]. UK medical colleges have further called for disinvestment in fossil fuels which include shale exploration because of the public health damage they do [11].

This research itself may not raise ethical or environmental justice concerns directly about shale, but the findings do and often for vulnerable groups and those living in already polluted communities or with pre-existing health problems. Effects range from the impacts of extracting materials to be used in fracking such as sand, the transport of materials and equipment to build, maintain and run sites as well as extract oil and gas, dispose of waste products and decommission wells and processing centres [78,79]. 

Associations between unconventional natural gas development in Marcellus shale and asthma exacerbation have been found [80,81]. The link between childhood hematologic cancer, air pollution and residential proximity to oil and gas development has been reported [82].

Associations between PM2.5 exposure and neurological disorders have been found in systematic reviews and meta-analyses [83,84,85]. Particular pollutants such as endocrine disruptors and other chemicals used and liberated in shale oil and gas present a range of air borne and water contamination hazards with risks that have not yet been fully quantified [86]. In such settings, the application of the precautionary principle would seem wise [87] and would help reflect ethical and environmental justice concerns in policy development.

Investigations of the effect of shale and birth outcomes in the USA were published in 2015 and later studies picked up concerns about very low-level effects on infant health [74,88,89,90,91,92].

Short-term exposure to air pollution and stroke have been linked in systematic reviews and meta-analyses [93,94]. In systematic reviews and meta-analyses, long-term exposure to PM2.5 was found to be an important risk factor for stroke [95]. 

The air quality pollutants identified by UK advisors during shale gas operations along with their sources are listed in Table 3 [96].

This picture is replicated in other assessments of some atmospheric pollutants in the shale exploration lifecycle [97].

There are still significant gaps: and in the US, they may be due to agencies at local and national levels pushing for cost and regulatory reasons against funding certain projects that address this deficit. For example, “direct measurements of key air pollutant emissions from unconventional oil and gas are limited, especially during drilling and completion (hydraulic fracturing and flowback) of new wells. Knowledge of emission rates of air toxics and other air pollutants from these activities is urgently needed to inform public policy” ([98], p. 720). In an evidence-led regulatory system, these gaps could be expected to trigger more precautionary policies [99,100]. Although there are very few detailed lifecycle assessments of fracking [101], there is enough information available to enable air pollution to be identified if not quantified in detail across the cycle. 

In assessing air pollution in fracking, deliberation tends to focus narrowly on planning and other laws relating to industry proposals for development of shale oil and gas. Wider and cumulative public health or environmental justice concerns are de facto ignored despite increasing recognition of the global impact of climate change and the role of the fossil fuel industry in contributing to that problem. 

### 3.2. Ethics and Health Impact Assessments (HIAs) of Shale Exploration

Ethical issues and questions of equity are important factors in HIA [102]. However, the HIA process, which often requires access to funding and expertise, can further exacerbate pre-existing inequalities. Shale oil and gas developments in areas of rural and industrial deprivation can reinforce pre-existing inequalities, where communities often experience relatively poor economic performance and health outcomes and may lack economic and cultural capital to contest planning applications. No independent regulatory body exists to assure the quality and equity of HIAs or control the conduct and practice of those carrying out HIAs within environmental statements or environmental impact statements. Guidelines have been drafted to address some of the identified deficits that have emerged but it is unclear the extent to which they have been applied. Key values that would underpin public health in HAI would centre on “democracy, equity, sustainable development and ethical use of evidence.” [103]. The International Association for Impact Assessment (IAIA) promotes a “guiding principle” for HIA, namely a “comprehensive approach to health” “HIA is most fundamentally concerned with the principles ‘do good and do not harm’. HIA provides a well-established approach to identifying both positive and negative impacts that may arise from a proposal” [42,43]. Again, there do not appear to be any public assessments of how environmental consultants in general and those working for the shale gas industry in particular apply these principles or have been audited to check their application. 

Shale oil and gas environmental impact statements, which include HIAs seldom, if ever, fully address sustainable development or environmental justice concerns. As such, they fail to reflect best practice. In addition, some shale exploration HIAs appear to rely on sweeping generalizations about risk and repeatedly cite studies which appear to downplay or neglect significant public health risks [104]. The IAIA endorses an integrated and participatory form of impact assessment. These are characterised by professional autonomy and transparency and adopt a broader view of social and health impacts. Yet many shale oil and gas HIAs appear to disregard community well-being and lack meaningful forms of participation. Often community consultation is tokenistic and does not address pre-existing information and power asymmetries skewed in favour of developers. This form of practice contravenes the IAIA acknowledged “duty of care to both present and future generations”. Yet shale oil and gas impact assessments fail to consider the global or local public health consequences of using shale energy [105,106,107].

The IAIA professional members are also expected to “not advance our private interests to the detriment of the public, our clients or employing institutions” How this guidance tallies with professional practice is unclear, particularly in circumstances where consultants’ reports are commissioned and financed by shale exploration companies [105]. The problems may be explicit or implicit in assessing, for example, the threats posed by air pollution in shale exploration planning proposals. This can be reflected in the language used by environmental consultancies in their reports: the terms selected and the standards presented may not accurately reflect either the importance of the public health agenda or the existing scientific evidence. Key threats to public health can be downplayed or ignored, including the failure to mention carcinogens, neurotoxins, immunotoxins or reproductive health hazards. The failure to reference the latest research findings is as commonplace as repeated references to narrowly drawn terms of reference or governmental standards which may not reflect the better public health standards available and certainly do not reflect the precautionary principle or the impact that socio-economic disadvantages have on vulnerable populations exposed to air pollution.

### 3.3. Inequalities and Environmental Justice in Shale Exploration

The geography of inequality linked to air pollution from conventional and unconventional gas extraction is also minimised or ignored by industry when presenting economic arguments for the expansion or continuation of oil and gas production [44,108]. Important analyses of shale oil and gas governance in the US linked to specific areas have been conducted and have much to offer in conceptualising the roles and relationships of different parties but the UK position is somewhat different. In the UK, there has been nothing like the equivalent research of ‘dumping down in Dixie’ or on first nations’ land, where the ethnicity of a local population affected by oil and gas air pollution was a key factor. In the UK, air pollution linked to shale exploration has been relatively under-researched but in the USA there is an evidence-base [109,110]. In the UK, inequality issues are not usually explicitly identified in papers addressing environmental justice and fracking [111] and may need wider parameters to include such elements as sustainability [112]. Public Health England, when estimating local mortality burdens due to air pollution, did not mention oil or gas specifically as contributors to those figures. Shale oil and gas commercial development at scale was not underway in the country at that time [38].

The geographies associated with inequality and air pollution suggest the impact on vulnerable populations, including workers, are simply missing from almost all industry and industrial consultants’ commentaries. Researchers have shown how industry

“Rebrands its flexible geographies—its variable spatial and temporal configuration of work, equipment, and bodies—as uncertain such that the industry, and its occupational exposures, are ungovernable. The evidentiary process of rulemaking has the effect of imbuing industry with ‘health’ such that human health risks are considered alongside industry’s capacities. Thus, the hearings provide a venue for industry to contest new regulations using discourses of uncertainty, not just regarding the science that justifies lower limits, but also the geographies on which the industry relies”.

[113,114]

The problems planners face with the fragmentation of projects and decision making is significant with regard to shale exploration [115]. It ensures the total picture of all shale oil and gas impacts is never fully considered and hence national and global burdens of air pollution that will affect small communities are not properly considered [116]. Comparisons by planners and planning appeals bodies of background air pollution levels in districts have also failed to fully consider fine particle pollution reported in the research.

Detailed work on socio-economic deprivation and impacts on public health linked to air pollution has been carried out in the US [117]. This has been relatively neglected in England. There is little research on the health effects of ultrafine particles [118,119]. In Scotland, there has been some effort by government to build these concerns into official deliberations on shale exploration, which reflect a more precautionary approach than that adopted by the UK government [107,120]. It is still an open question as to whether the more precautionary approach in Scotland is the product of a policy process receptive to the latest research or whether is reflects a pre-existing political preference of the Scottish government. Whatever the driver, our point is that this report illustrates how evidence, data, scientific consensus and uncertainty can be organised in and out of policy documents. Industry and industry-funded assessments of shale exploration air pollution impacts rarely address inequality or environmental justice concerns. In contrast, US researchers are beginning to factor in poverty and geography as well as many other influences relating to air pollution that would affect public health through cumulative environmental assessments affecting populations [114,121]. 

Similarly, other researchers are beginning to consider multiple environmental exposure risks and detailed lifecycle assessments from pre-conception to old age of individuals including their environment [122]. This is the exposome approach developed in 2005 but still neglected in the UK. It is another example of a lagging approach in England that has some validity to assessing possible impacts of air pollution along with a range of other factors [123,124,125].

In 2019, the UK government produced its Clean Air Strategy policy document under the umbrella of the Environment, Health, Business and Treasury ministries [126]. The strategy is unequivocal and, although not specifically written to address inequalities in health policy, it would benefit the most economically disadvantaged populations:

“Air quality is the largest environmental health risk in the UK. It shortens lives and contributes to chronic illness. Health can be affected both by short-term, high-pollution episodes and by long-term exposure to lower levels of pollution”.

“We will progressively cut public exposure to particulate matter pollution as suggested by the World Health Organization. We will set a new, ambitious, long-term target to reduce people’s exposure to PM2.5 and will publish evidence early in 2019 to examine what action would be needed to meet the WHO annual mean guideline limit of 10 μg/m^3^. By implementing the policies in this strategy, we will reduce PM2.5 concentrations across the UK, so that the number of people living in locations above the WHO guideline level of 10 μg/m^3^ is reduced by 50% by 2025”.

“We will equip health professionals to play a stronger role by working with the Medical Royal Colleges and the General Medical Council to embed air quality into the health professions’ education and training. We will work with local authorities and directors of public health to equip and enable them to lead and inform local decision-making to improve air quality more effectively”.

If this policy is adopted in England, it would help to address some of the environmental justice and ethical shortcomings created by proposed shale developments and climate change and air pollution impacts. Elsewhere in the world, fracking has been linked to human rights and clearly necessitates human rights impact assessments that would address environmental injustices [127]. In early 2020, it is still very unclear what progress will be made in reaching the WHO PM2.5 targets. The UK government’s own assessment of actions since January 2019 made in July 2019 failed to mention fossil fuels generally or shale gas in particular and shale exploration activities continued [9]. The UK in 2019 did meet the EU legislative limit value of 25 μg/m^3^ but had not met a second stage limit of 20 μg/m^3^ set for 2020. The UK limit itself is twice as high as the WHO guideline from 2008 and it did not meet the legal limits for NO_2_. 

### 3.4. Environmental Justice

Since the 1990s, those advocating environmental justice have made the connection between pollution, ethics and disadvantage. Fossil fuels and embodied energy injustices in coal have attracted attention [128]. The environmental justice advocates’ analysis remains valid in 2019 [129,130]. This moves the debate away from ‘simple’ or narrow risk assessments and locates it squarely in a wider ethical frame that is often entirely missed, even now, by UK policy makers and environmental consultants.

“The question of environmental justice is not anchored in a debate about whether or not decision makers should tinker with risk assessment and risk management. The environmental justice framework rests on developing tools and strategies to eliminate unfair, unjust, and inequitable conditions and decisions” ([131], p. 1).

The framework also attempts to examine the underlying assumptions that may contribute to and produce differential exposure and unequal protection. It brings to the surface the ethical and political questions of “who gets what, when, why, and how much” ([40], p. 559). In this context, work on the spatial analysis of populations and environments at risk from shale developments is especially important and plays directly into environmental justice concerns [112,132,133,134,135]. Proximity to natural gas wells in terms of exposures including air pollution may be associated in the US with prevalence of various health symptoms including respiratory illnesses in residents living nearby [136].

The health benefits of air pollution reduction are considerable. They are greatest in the most polluted areas but researchers note that they occur below international standards and play directly into reducing climate change impacts.

“Air pollution is a grave risk to human health that affects nearly everyone in the world and nearly every organ in the body. Fortunately, it is largely a preventable risk. Reducing pollution at its source can have a rapid and substantial impact on health. Within a few weeks, respiratory and irritation symptoms, such as shortness of breath, cough, phlegm, and sore throat, disappear; school absenteeism, clinic visits, hospitalizations, premature births, cardiovascular illness and death, and all-cause mortality decrease significantly” ([137], p. 1478).

So, cutting or preventing shale exploration is also an ethical step and will improve public health across the board along with reducing environmental injustices. The link between, for example, short-term exposure to fine particulate matter (particulate matter with diameter less than 2.5 μm (PM2.5)) and hospital admissions has been widely reported. Researchers conclude “new causes and previously identified causes of hospital admission associated with short term exposure to PM2.5 were found. These associations remained even at a daily PM2.5 concentration below the WHO 24-h guideline. Substantial economic costs were linked to a small increase in short term PM2.5” ([137], p. 1).

US and UK studies of fracking project proposals have identified major problems with planning and social licences to develop fracking and suggest power structures shape outcomes in favour of shale oil and gas schemes. There is little evidence that participation in enquiries on fracking by community members has direct impacts on decision making [105,111]. The lack of transparency, access to planning processes and decision making in UK energy developments such as shale exploration has also been well documented in recent years. Much of the focus recently has been on procedural injustice issues with less emphasis on problems of public health or ‘distributive’ injustice that had been covered in earlier years [41,111]. While it is true that without procedural justice in some settings, there will not be distributive justice, it is possible, as we have seen in parts of the US, France and Scotland, for government policy to prevent shale exploration at a national level, thereby ensuring no air pollution and no associated environmental injustice [107]. 

### 3.5. The Ethical Dimensions

The ethics of shale exploration and possible air pollution and climate change impacts have been generally neglected, with a few notable exceptions. In 2014, US researchers described the emphasis there on the cost–benefit analysis of shale, which of course includes climate change and public health. From an ethical perspective, they argued that policy makers had “a prima facie duty to minimize false negatives based on three considerations: (1) protection from serious harm generally takes precedence over the enhancement of welfare; (2) minimizing false negatives in this case is more respectful to people’s autonomy; and (3) alternative solutions exist that may provide many of the same benefits while minimizing many of the harms” ([32], p. 1114).

The oil and gas industry’s knowledge of the impacts of its products on the environment dates back almost a century, and its knowledge of the impacts of fossil fuels on the wider climate has been known since at least the 1980s. Grasso has identified five key ‘morally relevant’ facts about the industry, including air pollution and shale, in the context of addressing broader climate ethics.

“Oil and gas companies have known for decades that their activities caused climate change (Fact A—Awareness); they did not take steps to modify their fossil-fuel centred behaviour (Fact B—Behaviour), even though less carbon-intensive alternatives were possible (Fact C—Capacity). Additionally, oil and gas companies funded and orchestrated climate change denial campaigns, through which they successfully opposed political action against climate change (Fact D—Denial), while at the same time amassing and distributing fabulous wealth (Fact E—Enrichment) to the privileged few” [138].

As Table 4 below shows, in the UK, there was often little consideration of ethical, environmental justice or equality issues when examining the possibilities of shale exploration and potential impacts on communities and the climate [139,140,141,142,143,144,145,146,147]. In contrast, there was much discussion of planning technicalities. This is somewhat surprising because, for example, Public Health England’s role specifically included addressing health inequalities. By contrast, Health Protection Scotland [64] did address a number of these issues in a report (with a similar brief to that produced by PHE) and looked in detail at health inequalities. The Smith reports [142,143,144,145,146], although given much prominence in the media and much weight by industry and the UK government, were funded by industry and produced superficial analyses in support of the shale industry while largely neglecting public health research.

The two House of Commons research briefings for MPs prepared in 2015 and 2018 flagged planning issues but ignored environmental justice, inequalities and ethical dimensions of shale exploration. These reports also made little or no mention of air pollution and shale beyond brief reference to traffic impacts but they did flag climate issues. The UK government position prior to the 2019 election reflected in its guidance was that fracking in the UK had measures in place to ensure on-site safety, prevent environmental contamination, mitigate seismic activity and minimise greenhouse gas emissions [37]. Hence, the UK government policy did not recognise any adverse climate change or air pollution health effects from shale exploration and did not acknowledge any environmental justice, inequalities or ethical issues raised by shale oil and gas. In November 2019, with an election looming, the government changed its position and announced a moratorium on shale gas development in England. This appeared to be an attempt in the short term to avoid alienating its electoral base in rural areas that usually supported the Conservative government. The government argued it adopted a precautionary and sustainable approach to shale prior to this date, geared towards minimising disturbance to those living and working near to shale developments and preventing risk of any damage [140]. However, given this rather dramatic policy shift it is therefore difficult to explain why the government does not adopt the WHO standards on air pollution from sources that would have included shale. 

A critique of environmental impact assessments and their ethical deficiencies remains that they are too narrow and focussed on single issues (e.g., sustainability, utility or safety) [149]. It could, however, be argued that EIA practitioners when dealing with shale exploration proposals are not sufficiently focussed on climate change threats and because they deal with relatively small projects, underplay the threats to global public health from the sector as a whole.

These decisions do not exist in a vacuum. The relationships especially in the USA between lawmakers and oil and chemical companies, for example, to influence policy makers have been detailed over many years [2,3] and they have a major effect on environmental justice. In the UK, NGOs have explored and described such shale industry relationships but academic studies in the UK have neglected this important aspect of the issue culture [150].

Ethical concerns about environmental impact assessments have been raised since at least the early 1980s in professional journals [102]. “Ethical Impact Assessment (eIA) is an approach for contextualized ethical assessment of technology development projects by developers and decision makers involved in the project” ([151], p. 1). Impacts would include those “that concern or affect rights and responsibilities, benefits and harms, justice”. 

Guidelines do exist that could address some of the ethical dilemmas that arise although in the shale gas field it would seem consultancy companies do not necessarily recognise them or appear to act on them [105]. 

## 4. A Typology of Industry and Consultancy Approaches to Shale Developments in England: Lessons on Air Pollution and Public Health (Drawn from Analyses of Proposals and Related Regulatory Commentaries and Reports) 

The synthesis developed here brings together a typology of arguments used to justify neglect of air pollution, ethics and environmental justice and some evidence-based rebuttals [105,106,107,152]. We have structured this discussion by first identifying the prevailing ‘common sense’ in policy making, and then discussing the assumptions and evidence that are organized in and out of policy discourse. This allows us to set up a counter-factual heuristic that draws attention to evidence that can be used to challenge policy making ‘common sense’.

### 4.1. Planning Design

The prevailing official, industry and industry consultant view is that the English planning system is capable of assessing shale exploration risks linked to air pollution.

This is contested. The English planning system, by current design, cannot effectively consider the cumulative effects of developments in a sector where shale exploration may be developed at scale and multiple shale wells may be opened up. In practice, each proposal is considered separately. One defence for the status quo could be that air quality in any area is considered with each application. However, this approach is retrospective and the planning system as currently configured struggles to assess multiple applications and their cumulative effects. The Institute of Air Quality Management and Environmental Protection UK guidance for the consideration of air quality within the land-use planning and development control processes in January 2017 states:

“As a minimum, the planning system should not take decisions on individual proposals that lead to unacceptably poor air quality, nor should it make a series of decisions that collectively produces this undesirable outcome” ([153], p. 9).

Local authorities in England have grappled with the problem of knowing large scale shale explorations are planned. Given the constraints of current planning legislation, authorities are required to make determinations on only specific proposals. The professional air quality institution in the UK recognised this problem in its guidance:

“A particular concern of many local authorities is that individual developments are often shown to have a very small air quality impact, and, as a consequence, there are few mechanisms available to the planning officer to require the developer to achieve lower emissions. This, in turn, leads to concerns about the potential air quality impacts of cumulative developments as many individual schemes, deemed insignificant in themselves, contribute to a ‘creeping baseline’” ([153], p. 16).

### 4.2. Planning Equity

The operational consensus among planning officials, industry and industry consultants on air pollution is that equity exists in the planning system for shale proposals. The assumption is that, in principle, adequate resources are available to all sides and the use of comprehensive impact assessments to assess such proposals is equitable.

This can be contested. The problem has been compounded by the system, whereby English local authorities may turn down shale projects only to find their decisions overturned in planning appeals or by central government. This raises questions about the social licence to exploit shale. The imposition of shale developments on communities where the local democratic means of scrutinising and approving such plans are effectively vetoed raises profound questions of ethics and environmental justice. In addition, the cumulative air pollution impact assessments of shale exploration in England lag behind the science. Relatively little attention has been paid by industry or its consultants to full cumulative environmental impact assessments or exposome calculations of risk from air and other pollutants [117,121,122,125,154,155,156]. The exposome concept developed in 2005 examines total environmental exposures during a life time to a range of external factors [78] and more targeted research focusing on critical and sensitive windows of human development is adding to our knowledge of the complex interactions between environmental exposures and health over the lifespan [157].

The lag between publication of scientific research and its inclusion in planning deliberations is further compounded by a planning system that does not require input from or oversight by public health professionals. In several enquiries, only when proposals had been considered, approved or contested, were public health professionals able to comment on shale exploration.

No full public health or environmental lifecycle analysis are provided by industry or their air quality consultants when proposing shale exploration. This is despite calls for their application along with chemical usage and risk assessment work [78] and from the UK government shale gas air quality expert group [96]. The expert group acknowledge that “in order to enable evaluation of the impact on local air quality, a full well lifecycle analysis is required for a range of pollutants relevant for a range of issues including health, and agricultural and natural ecosystems” ([96], p. 11). A Spanish pilot LCA has noted how limited data are on the industry and stressed the need for a precautionary policy [101]. Other research that included air pollution and poor air quality considerations noted the UK lack of precaution in shale exploration with regard to public health [105,106,107,158]. LCAs are not currently legally required in shale development submissions. This approach precludes a comprehensive air quality assessment and constrains the parameters of any consultant assessments. Consultants heavily rely on information that can only be provided by private companies involved in shale oil and gas extraction. While consultants also usually draw on data from other sources to make assessments there are inherent weaknesses associated with current practice, not least the lag in adopting lifecycle analyses, the underplaying of precaution and arguably the overstatement of the health of shale proposals. 

### 4.3. Data and Evidence: Health

The prevailing policy making orthodoxy is that sufficient air quality research and information are available to evidence the health and safety of proposals in process and outcomes

This can be contested. The planning and approval process does not test information acquired and can ignore recent research in producing hypothetical risk models and predictions. There appears to be little interest in planning governance to test whether information provided by companies involved in shale developments is reliable or accurate. If all the evidence is available, then it can be fully assessed and used in an air quality assessment or discarded as not relevant or of minor significance if that is the case. 

One of the particular challenges facing commercial consultants working in this field is to ensure transparency. They should be able to demonstrate that risk assessments are based on the latest public health research as well as recognising where important gaps may exist. This means not simply claiming reports are based on the latest research (which most do) but citing sources other than those from government and the industry itself about hazards and risks (which most commercial consultants do not). The DEFRA report from the air quality expert group on shale exploration, completed in 2015 drawing on the latest research available at that date, illustrates why this is so important. It uses data from the latest US studies, recognizes where data gaps exist, and applies lifecycle analyses to shale gas extraction. This stands in marked contrast to other expert inputs in planning processes on shale in England. 

Acute effects of air pollution are often neglected in shale planning applications. Air quality cannot be examined effectively without reference to the diseases that poor air quality may cause or contribute to. The effects of both short-term and low-level exposure to PM2.5 and ozone including asthma has been documented [159,160]. IAQM/EPUK guidance in 2017 observed that “It is likely that removing exposure to all PM2.5 would have a bigger impact on life expectancy in England and Wales than eliminating passive smoking or road traffic accidents” ([153], p. 9) and refers to the importance of a coherent strategy underpinning air quality management. This would appear to support strategic decision-making on air quality by local authorities geared towards reducing PM2.5 loads rather than continuing with the current system of largely piecemeal judgements on individual industry planning applications.

Both PM2.5 and PM10 were positively associated with admission to hospital for stroke or mortality from stroke, with a stronger association for PM2.5 [93]. A brief increase in airborne fine particulate matter PM2.5 is also associated with the development of acute lower respiratory infection in young children [161]. Shale development has also been associated with increased hospital utilization rates generally [162]. ‘Diesel’, a source of PM, in planning proposals for shale is often not mentioned either in terms of its carcinogenicity or other possible adverse health effects related to exposure to its emissions [79]. Diesel pollution from shale exploration site transport and operations has been flagged as an issue for workers and communities [163]. One recent UK study suggests that exposure to diesel engine exhaust emissions from shale equipment could present a significant risk to people working on such sites over extended time periods [79]. Failure to identify all sources of diesel exposure for the exposed population would be a serious omission from a public health perspective.

‘Respiratory’ illnesses including asthma are also rarely mentioned in English shale proposals by some industry environmental consultants. There are usually no specific disease references to diesel engine exhaust emissions (DEEE), listed by the WHO International Agency for Research on Cancer in 2012 as a group 1 known human carcinogen with lung cancer and a probable bladder cancer association [164]. In the context of cumulative exposures to some individuals from a range of environmental and occupational sources, this is again an environmental justice and ethical issue, suggesting both lagging and flagging responses from industry and government.

### 4.4. Data and Evidence: Environment

There is also a view among planners, industry and commercial consultants that sufficient air quality research and information are available to demonstrate the safety of proposals in terms of environmental justice, including vulnerable populations.

This can be contested. Industry and its consultants often fail to mention explicitly ‘vulnerable’ populations in their documents supporting shale developments. There is no official standard on what acceptable air pollution limits for such populations might be. For the World Health Organization and public health professionals, such groups are of special concern and may be more susceptible to the adverse effects of poor air quality even in the short term and at low levels of exposure than other groups. According to Dr Bustreo from the WHO, “air pollution continues take a toll on the health of the most vulnerable populations—women, children and the older adults. For people to be healthy, they must breathe clean air from their first breath to their last” [165]. To find no discussion of the position of this group in shale exploration proposals is a major omission from a public health perspective. Yet studies have looked at prenatal and perinatal exposures linked to unconventional oil and gas operations that also raise questions about possible cross-generational effects of air pollutants [166,167].

A leading US public health physician (who looked at a range of chemicals including benzene) came to the following stark conclusion. “By sharply reducing our dependence on fossil fuels we would achieve highly significant health and economic benefits for our children and their future. These benefits would occur immediately and also play out over the life course and potentially across generations” [168].

“Subjects with chronic respiratory diseases such as chronic obstructive pulmonary disease (COPD) and asthma are especially vulnerable to the detrimental effects of air pollutants” ([169], p. 1). The UK NHS recognises air pollution as a trigger for asthma [170]. So, if air quality declines even for a short period such as a few months, then populations could be adversely affected especially those proximate to the sources of air pollution.

One argument has been that buffer zones (setbacks) for shale developments will protect these populations from air pollution and are thereby both environmentally just and ethical. However, researchers exploring air pollution and other risks suggest “setbacks may not be sufficient to reduce potential threats to human health in areas where hydraulic fracturing occurs. It is more likely that a combination of reasonable setbacks with controls for other sources of pollution associated with the process will be required” ([171], p. 1323). They further noted “unfortunately, there is no defined setback distance that assures safety” ([171], p. 1330). 

A later US air pollution study from 2018 of working wells found that even though the setback distance policy in their state (Pennsylvania of 500 ft. or 152.4 m) might be effective in some cases, exposure limit exceedance occurred frequently at this distance with higher than average emission rates and/or greater number of wells per well pad. Existing standards were viewed as inadequate [172]. Some US studies have called for additional setbacks where vulnerable groups are found, including schools, day care centres, and hospitals. They also agreed that setback distances should always be greater than ¼ mile (402 m) and some argued for 2 mile set-back zones [173]. Again, there is evidence of lagging policy making despite the latest research raising key concerns.

### 4.5. Sustainability

Until very recently, government policy and regulations suggested that climate change and sustainability linked to air pollution were either marginal or irrelevant to shale exploration proposals. This approach is increasingly contested. Until 2019, there was little interest by government in addressing the climate change effects of developments. However, in early 2019, a shale exploration enquiry in England was the first of its kind to consider the impact of onshore oil and gas on climate change. The Planning Inspectorate in June 2019 then indicated the decision would be delayed on the project following the publication of greenhouse gas reduction targets by the government’s advisor on climate change [174]. The Committee on Climate Change in May 2019 indicated the UK should phase out greenhouse gas emissions by 2050. The report recommended that a net zero target should be put into law as soon as possible, but did not specifically mention shale exploration [175]. Whether the target will affect this and other planning decisions is not yet clear. However, climate change due to fossil fuels and materials used in their extraction will adversely affect air quality globally and locally.

## 5. Discussion

The science on climate change, air pollution and shale exploration is clear. In the UK, debate focuses on the scale of the impact and the weighting of effects from other sources of air pollution. However, in the day-to-day practical and operational assumptions of those professionally involved in planning and regulation related to shale (officials, developers and their hired consultants), it is difficult to discern how the latest science, and the policy guidance available to planners and experts, actually and effectively positively influence policy and planning decisions from a public health perspective. Additionally, research is often funded by both industry and government and government scientific advisors have all too often ignored international research on climate change and air pollution and rarely touch on ethical and environmental justice considerations and concerns in their assessment of shale exploration. The impact of industry on government policy in England in this field has been considerable and worked its way through both energy and planning policy at a national level but not necessarily local level through public health structures. Environmental justice and ethics elements are effectively absent from governance and decision making. Electors—in the UK context, this refers to those who elect local and central government MPs and councillors and also community council members—may be concerned about environmental justice and seek ethical support for their concerns about shale exploration. All too often those concerns are either superficially noted, marginalized or ignored by government (which, in our conceptualisation, includes regulators and planning officials) and industry at the policy and planning levels as Diagram1 showed. 

Industry has been able to effectively defend its position by recourse to established ‘common sense’ views about how the economy should operate, a common sense carefully crafted in policy planning groups and policy networks by business leaders and ideologues [176,177]. As such, the devotion of politicians to growth and job creation, to short-term decision making, all reflects the hegemonic grip of market-friendly ideas on those involved in policy. In these terms, it may well appear as a ‘common sense’ that shale oil and gas jobs and economic benefits are a self-evident good. Those who already have an ideological affinity with market-friendly ideals may well believe that the alleged social benefits from shale developments will arise. Other interested parties such as regulators, planners, the police in several instances, and environmental consultants may also believe in these projections, or at least comply with decision making that does not robustly query or dispute such claims. As we have tried to demonstrate in some detail above, these beliefs are only possible if you either distrust or ignore the scientific evidence and if you do not seriously weigh ethical and environmental justice concerns in decision making.

This links to how industry can “cement and unify diverse social interests and aspirations” [45] but such actions are about promoting private interests (sometimes in the guise of common interests, like ‘shale exploration is good for UK plc’) to the detriment of public health and environmental sustainability. It is something of a truism in policy analysis that narrow business interests often triumph in lobbying and influencing strategies partly because it is easier to mobilise around narrow sectional interests and partly because the opposition may be diffuse and relatively disorganised. Who speaks for marginalised and vulnerable populations, and what do they say? The industry therefore adopts a range of measures to protect its privilege. Business is a key stakeholder for government and is regularly consulted on policy. New policy proposals are subjected to market testing, and business cases need to be made for social investment. Thus, market logics are a form of dominant ideology or orthodoxy that profoundly shape all sorts of policy fields, including those of public health and environmental protection. Factions in civil society resist actions that may damage their local, regional, national and international public health and environment by opposing projects (such as using legal and direct-action campaigns). 

However, there are other factions in civil society who actively promote development. The movements for ecological justice and sustainability may have more members, but the counter movements who deny or dismiss climate change have powerful backers and part of the contestation of these issues takes place on the terrain of civil society [47,51,52,178,179]. Civil society, in this conceptualisation, includes grassroot movements, campaign groups and indeed professional groups (including associations of planners, health experts, and environmental consultants) as well as business associations and lobby groups. Struggles over policies that protect public health and the environment take place across civil society and are linked to the technocratic and deliberative fora of planning tribunals and regulatory processes. Public health experts, environmental consultants and planning officials are deeply embedded in these debates, as they play out in public via mass and social media, and in more private settings like official hearings, tribunals and advisory committees and working groups. How their participation in these is shaped by their own value commitments, as well as professional norms and guidance produced by professional bodies is a key question that may repay further debate and examination as the stakes around policy making on climate and public health are raised. 

The critique we have developed in this paper asserts that the deliberative practices and regulatory mechanisms that exist to promote economic growth, protect the environment and promote public health are all thoroughly shot through with normative assumptions that are often in competition with each other. The politics of this topic require decision makers to try to resolve these contradictions. The repeated rhetorical commitment by government and industry to regulatory best practice and protecting public health is difficult to sustain where regulatory capacity cannot ensure best practice or secure public health. There appears to be consensus among public health scientists towards adopting more precautionary approaches.

## 6. Conclusions

National and international decision making on developments marginalised, downplayed or ignored the science and public health concerns about shale exploration and latterly specifically air pollution. The effects of conventional and unconventional oil and gas would seem to demonstrate that the shale oil and gas industry position is effectively unscientific. The shale industry’s defence in England of its activities has been very effective, despite some delays in terms of progressing through planning applications and, where these were refused, appealing them and gaining government support for developments.

The industry in England has largely operated in a policy field where the core assumptions of planners and decision makers have quite clearly been in favour of shale development at scale. This state of affairs was unexpectedly disrupted in November 2019, when an English shale moratorium was introduced. However, despite this policy reversal (in the context of a general election campaign and with the pretext of the seismic impact of shale exploration rather than on grounds of climate or public health), major air pollution and associated climate change threats remain ‘denied’ and either wholly or partially unaddressed and so ethical and environmental justice dimensions of air pollution have been ignored and marginalised. Hence, the lagging structure for dealing with new shale exploration in England continues and the country lags behind the science and international initiatives on climate change and air pollution. 

Global solutions to address the problems of climate change which impact on air pollution problems have been widely mooted [180] but action by UK national and local government bodies and some of their scientific advisors as well as professional bodies remains constrained. The scientific evidence can be ignored in planning proposals and health impact assessments through a variety of mechanisms, that are both explicit and implicit. By ignoring or downplaying the science, public health is damaged and industry and its consultants privileged [181]. Other analyses of shale extraction, policy and governance throw up some similar but also different relationships and different models. For example, urban drilling occurs in Texas and may shape relationships and responses to shale exploration and setbacks but there is no urban drilling in England and the relationships of local government and civil society with firms in the UK are quite different [182]. We therefore offer an analysis that does not always fit with US and other models and does pick up in broad-brush terms the questions of ethics and environmental justice linked to climate change and air pollution.

Any new policies should ensure that there are ethical approaches to proposed shale exploration that if not capable of ensuring environmental justice will at least bring it much closer in the 2020s. England remains lagging and flagging behind both Scotland, for example, and the science in combatting air pollution and climate change from fossil fuels and shale.

## Figures and Tables

**Table 2:** Characterisation of the main sources of air pollution from oil and gas development according to well process stage [22]. Source NRDC. Used with permission.

Emissions Source	Local							Regional		Global	
	Particulate Matter (Pm)		Volatile Organic Compounds (Vocs)			H_2_s	Respirable Silica	Vocs	no_x_	Greenhouse Gases	
	Diesel Pm	Pm_10_	bTex	PAH (Incl. Naphthalene, Chlorobenzene, Phenol)	Other (Incl. Formaldehyde, Ethylene Glycol, Methanol)					cH_4_	co_2_
**Well site preparation (landscape clearing, soil** **movement,** **pipelines and other infrastructure)**	**•**	**•**	**•**	•					•		•
Well drilling, hydraulic fracturing and well completion drill rig, drilling muds and cuttings, fracturing fluid mixing, water trucks, pumps, generators, flowback	•	•	•	•	•	•	•	•	•	•	•
Well production (produced water, gas flaring/ venting, well maintenance work)	•	•	•	•	•	•		•	•	•	•
processing and storage (gas venting, glycol dehydrators, separators, condensate tanks, compressors)	•	•	•	•	•	•		•	•	•	•
Transmission (compressors, gas venting, pipelines, tanker trucks	•	•	•	•	•			•	•	•	•
Well abandonment & site rehabilitation	•	•	•	•					•	•	•

*Key:* BTEX: benzene, toluene, ethylbenzene, xylene; CH_4_: methane; CO_2_: carbon dioxide; diesel pM: diesel particulate matter; H_2_S: hydrogen sulfide; NO_x_: nitrogen oxides; O_3_: ozone; paH: polycyclic aromatic hydrocarbons; pM_10_: particulate matter of 10 micrometers or smaller in diameter.

**Table 3:** Air Quality pollutants from shale gas operations.

Pollutant	Source	Potential Consequences
Non-methane volatile organic compounds	Fugitive emissions from drilling, during the extraction process, storage, venting and capped wells. Flaring and use of mobile machinery	0_3_ formation and health impacts
Nitrogen oxides	Flaring, mobile machinery, usage, gas processing, freight vehicles	0_3_ formation and health impacts
Methane	Leakage from well exploration, extraction and abandonment activities	0_3_ formation
Particulate matter	Suspension of bulk materials handling, flaring, mobile machinery, suspension/resuspension from freight vehicles	Health impacts
Sulphur compounds	Drilling, flowback phase and flaring	Health impacts and odour nuisance

Source: Air quality expert group to the Department for Environment, Food and Rural Affairs; Scottish government; Welsh government; and Department of the Environment in Northern Ireland. Potential air quality impacts of shale gas extraction in the UK. 2018:20 [96].

**Table 4:** Environmental justice, ethics and planning issues cited in UK shale oil and gas reports: number of times these topics are mentioned in the listed papers and reports.

Date	Report Author	Body	Environmental Justice	Inequalities	Ethics	Planning
2012	Royal Society Royal Academy of Engineering [139]	Report for Government	0	0	0	40
2014	Public Health England [38]	Government agency	1	0	1	47
2014	Independent Expert Panel. Scotland [133]	Report for Scottish Government	2	0	1	57
2015	House of Commons shale gas. White et al. [142]	Parliamentary Research Briefing	0	0	0	48
2015	Task force on Shale (Smith): Final Conclusions [143]	Industry funded	0	0	0	3
“	4th interim report economic [144]	Industry funded	0	0	0	6
“	3rd interim report. Climate change [145]	Industry funded	0	0	0	4
“	2nd Interim report. Environment and health [146]	Industry funded	0	0	0	13
“	1st interim report. Planning, Regulation and Local Engagement [147]	Industry funded	0	0	0	106
2016	Health Protection Scotland. Full report [64]	Scottish Government Agency	1	21	0	97
2016	Saunders et al. [76]	Academic paper	2	0	2	9
2018	House of Commons shale gas. Priestley and Hinson [148]	Parliamentary Research briefing	0	0	0	100
2019	DBEIS UK Government Guidance on fracking development [37]	Government department	0	0	0	16
